# Dual Super-Systolic Core for Real-Time Reconstructive Algorithms of High-Resolution Radar/SAR Imaging Systems

**DOI:** 10.3390/s120302539

**Published:** 2012-02-24

**Authors:** Alejandro Castillo Atoche, Javier Vázquez Castillo

**Affiliations:** 1 Department of Mechatronics, Autonomous University of Yucatan, Av. Industrias No Contaminantes s/n, Cordemex, 97203, Merida, Yuc., Mexico; 2 Science and Engineering Division, University of Quintana Roo, Boulevard Bahia s/n, Chetumal, QRoo 77010, Mexico; E-Mail: jvazquez@uqroo.mx

**Keywords:** super-systolic, parallel computing, remote sensing, FPGA

## Abstract

A high-speed dual super-systolic core for reconstructive signal processing (SP) operations consists of a double parallel systolic array (SA) machine in which each processing element of the array is also conceptualized as another SA in a bit-level fashion. In this study, we addressed the design of a high-speed dual super-systolic array (SSA) core for the enhancement/reconstruction of remote sensing (RS) imaging of radar/synthetic aperture radar (SAR) sensor systems. The selected reconstructive SP algorithms are efficiently transformed in their parallel representation and then, they are mapped into an efficient high performance embedded computing (HPEC) architecture in reconfigurable Xilinx field programmable gate array (FPGA) platforms. As an implementation test case, the proposed approach was aggregated in a HW/SW co-design scheme in order to solve the nonlinear ill-posed inverse problem of nonparametric estimation of the power spatial spectrum pattern (SSP) from a remotely sensed scene. We show how such dual SSA core, drastically reduces the computational load of complex RS regularization techniques achieving the required real-time operational mode.

## 1. Introduction

Advances in digital signal processing have permeated many applications, providing unprecedented growth in capabilities. Complex sensors systems are computationally extremely expensive and the majority of them are not suitable for a real-time implementation [1–20]. Through the advent of programmable computing, many of these processing remote sensing (RS) algorithms have been implemented in more general-purpose computing while still preserving compute-intensive functions in dedicated hardware.

Moreover, a mix of dedicated hardware solutions and programmable devices is found in applications for which no other approach can meet their real-time performance demands. Additionally, many hyperspectral imaging applications require a response in real-time in several areas for example, environmental modeling and assessment, target detection for military and homeland defense/security purposes, and risk prevention and response. Even though newer microprocessors can operate at several GHz in speed providing a maximum throughput in the gigaflops class, several contemporary applications such as space systems, airborne systems, missile seekers, tracking wildfires, detecting biological threats, and monitoring oil spills, to name a few, must rely on a combination of dedicated hardware and programmable embedded systems. As a result of the growth in the requisite parallel data processing, high-performance embedded computing (HPEC) architectures represent a real possible solution in order to achieve a high performance in real time implementations, especially in those applications that include complex RS reconstructive operations.

The main contribution of this paper is the design of an efficient high-speed dual super-systolic array (SSA) core for reconstructive signal processing (SP) operations of RS algorithms via the use of HPEC techniques. With the design of a bit-level dual SSA core architecture, the computational load of complex RS algorithms can be drastically reduced pursuing the required real-time operational mode for newer Geospatial applications.

The strategy for the implementation of a bit-level dual super-systolic architecture in a Xilinx Virtex field programmable gate array (FPGA) platform is aimed at enhancing the locality through the utilization of HPEC techniques to precisely represent loop programs and computing complex sequences of loop transformations (interchange, skewing, tiling, *etc*.) while preserving the original program semantics and also, by mapping the transformed algorithmic representation in SSAs. Likewise, this transformation performs the hardware projections in a bit-level parallel fashion. It is important to remark that with the combination of different hardware scheduler and allocation functions other SSA’s architectures can be implemented.

Therefore, although there are some recently developed studies related to the implementation of RS applications [6–20], there still remain some unresolved implementation issues related to the efficient hardware level implementation of multi-processor system-on-chip (MPSoC) architectures. Typical sequential implementations of these RS algorithms are traditionally implemented in synthetic aperture radar (SAR) simulations scenarios with model uncertainties previously developed by [6,7]. Moreover, the use of novel parallel computing techniques applied in the design of the proposed SSAs will allow the maximum possible of parallelism and the best performance implementation than software simulations and traditional HW-level architectures provided in other studies [8–11,16–19,21]. For example, a HW/SW co-design method was developed in [17], in order to achieve the near real time implementation of the convex regularization-based procedures for reconstructive signal processing operations. However, in that previous study, no SSA architectures were considered. In [16], an approach with the incorporation of SA-based implementation schemes is proposed, but the architecture is reduced only to the matched space filtering based on the triple matrix multiplication and the 1-D convolution. In [21], a bit-level high-speed VLSI architecture of a matrix-vector using multiple arrays of processors in aggregation with the HW/SW co-design is presented. In the study, only the main concepts of a SSA architecture are described and a simple bit-level matrix-vector structure is presented without detail.

Finally in Section 4.2 of this study, a test case study of how the dual SSA core drastically reduces the processing time of a high-resolution regularization technique for the enhancement/reconstruction of real world SAR images is presented. Such hardware implementation results illustrate the usefulness for the development of system-level optimization of high-resolution image enhancement tasks performed with real-world RS imagery. The authors believe that the proposed high-speed dual super-systolic core architecture is *unique* and *differs completely* from the approaches of the recently developed studies discussed above. Additionally, with the relevant bit-level dual-core architecture based on SSAs, a new paradigm related to the design of a specialized HPEC hardware module is introduced.

## 2. Design Flow

**Figure 1.**
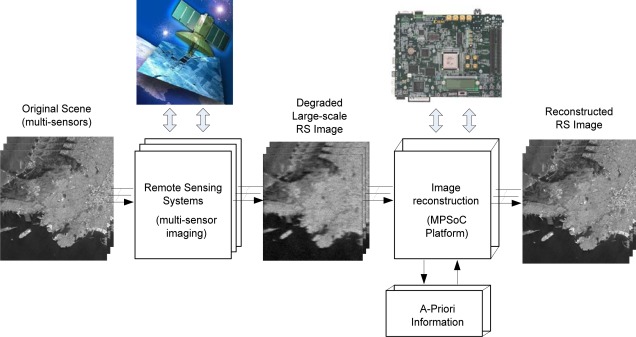
MPSoC platform of RS algorithms via the HW/SW co-design paradigm.

The term remote sensing (RS) is used to describe the science of identifying, observing, and measuring an object without coming into direct contact with it. This process involves the detection and measurement of different wavelength radiations, reflected or emitted from distant objects or materials, by which they may be identified and categorized by class, type, substance, and spatial distribution. RS systems are thus made of sensors mounted on an aircraft or a spacecraft that gather information from the Earth’s surface. Synthetic Aperture Radar (SAR) is an array of active sensors, and it is widely used in remote sensing missions to achieve high-resolution Earth images. In recent years, several efforts have been directed towards the incorporation of high-performance computing (HPC) models to remote sensing missions. Moreover, advances in sensor technology are revolutionizing the way remotely sensed data are collected, managed, and analyzed. In particular, many current and future applications of remote sensing in earth science, space science, and soon in exploration science require real- or near-real-time processing capabilities. In Figure 1, a multi-sensor image acquisition and reconstructive processing system based on a MPSoC platform for the enhancement/reconstruction of RS algorithms via the HW/SW co-design paradigm is illustrated.

In this study, we also propose a design methodology for real time implementation of specialized arrays of processors in a high performance embedded computing (HPEC) architecture. This architecture is based on a dual super-systolic array core as coprocessors unit that is integrated in a MPSoC platform via a HW/SW co-design paradigm.

This approach represents a real possibility for high-speed reconstructive signal processing (SP) tasks for the enhancement/reconstruction of RS imagery. In addition, the authors believe that the FPGA/DSP-based systems in aggregation with novel bit-level super-systolic architectures are emerging as newer solutions which offer enormous computation potential in RS systems.

### 2.1. HW/SW Co-Design Methodology

In this sub-section, we describe the HW/SW co-design methodology implemented in this study. The HW/SW co-design is a hybrid method aimed at increasing the flexibility of the implementation and improvement of the overall design process [16–20]. When a co-processor-based solution is employed in the HW/SW co-design architecture, the computational time can be drastically reduced. Two opposite alternatives can be considered when exploring the HW/SW co-design of a complex SP system. One of them is the use of standard components whose functionality can be defined by means of programming. The other one is the implementation of this functionality via a microelectronic circuit specifically tailored for that application. It is well known that the first alternative (the software alternative) provides solutions that present a great flexibility in spite of high area requirements and long execution times, while the second one (the hardware alternative) optimizes the size aspects and the operation speed but limits the flexibility of the solution. Halfway between both, hardware/software co-design techniques try to obtain an appropriate trade-off between the advantages and drawbacks of these two approaches.

The HW/SW co-design methodology encompasses the following general stages:

- Algorithmic implementation (reference simulation in MATLAB and C++ platforms);
- Computational partitioning process;
- Architecture design procedure using HPEC techniques.

From the analysis of the HW/SW co-design methodology, one can deduce that the RS algorithm is first adapted in a co-design scheme applying HPEC techniques, and then, the selected computationally complex reconstructive operations are efficiently implemented in bit-level high-throughput accelerator architectures.

#### 2.1.1. Algorithmic Implementation Analysis

In this sub-section, the procedure for the computational implementation of the RS-related regularization algorithms using MATLAB and C++ platforms is developed. With these algorithmic analyses, the effectiveness of the model employed in the HW/SW co-design is verified.

All the numerical test sequences are generated with the Fixed Point Toolbox [22] of MATLAB in order to verify computationally the proposed HW/SW co-design methodology (*i.e.*, test sequences for performing the SW simulation and for the HW verifications). Also, the Minimum Square Error (MSE) test is implemented to verify the correct fixed-point implementation (*i.e.*, for signed numbers in two’s complement format). In the case of C++ platform, this analysis is performing in order to evaluate the real-time performance analysis. The results of such SW simulation and HW performance analysis will be presented and discussed further on in Sections 4.1 and 4.2.

Now, we briefly describe a family of previously developed nonparametric high-resolution RS imaging techniques [12,13,15–20], via the generalization of their regularization optimization formalism. Such techniques incorporate different regularization and computation paradigms that enable one to modify some controllable algorithmic-level “degrees of freedom” as well as design a variety of efficient aggregated/fused data/image processing methods.

Examples of different RS imaging methods are the following: the Constrained Least Squares (CLS) and the Weighted CLS, which are deterministic methods that incorporate partial error functions into the corresponding objective costs [20]. In [12,13], the unified descriptive experiment design regularization (DEDR) paradigm incorporates into the unified optimization problem, other robust and more sophisticated statistical methods, among them are: the rough conventional matched spatial filtering (MSF) approach [3]; the descriptive maximum entropy (ME) technique [20]; the robust spatial filtering (RSF) method [12], the robust adaptive spatial filtering (RASF) technique [13], the fused Bayesian-DEDR regularization (FBR) method [20]; *etc*. All such DEDR optimization procedures have been detailed in previous studies [12,13,16–20]. It is important to remark that due to the non-linearity of the objective functions, the solution of the parametrically controlled fusion-optimization problem will require extremely complex no-parametric algorithms [20] and result in a technically intractable computational schemes if solve these problems employing the standard simulation software and hardware platforms based on DSPs and networks of CPUs.

The above implementation schemes are optimized in order to solve RS imaging problems, stated as follows: the scene pixel-frame image **B̂** is estimated via lexicographical reordering **B̂** = *L*{**b̂**} of the spatial spectrum pattern (SSP) vector **b̂** reconstructed from whatever available measurements of independent realizations {**u***_(j)_*; *j* = 1, …, *J*} of the recorded data vector. Thus, one can seek to find, **b̂**, as a discrete-form representation of the desired SSP, given the data correlation matrix **R_u_** = **Y** pre-estimated empirically via averaging *J* ≥ 1 recorded data vector snapshots {**u***_(j)_*} [12]; and by determining the solution operator that we also refer to as the signal formation operator (SFO) **F** such that:

(1) $$\begin{array}{l} {\hat{b}}^{\left(p\right)}={\left\{{\hat{R}}_{e}\right\}}_{\text{diag}}={\left\{F^{\left(p\right)}{\mathrm{YF}}^{(p)+}\right\}}_{\text{diag}}={\left(F^{\left(p\right)}{\mathrm{uu}}^{+}F^{(p)+}\right)}_{\text{diag}}\\ =(F^{\left(p\right)}u)⊙\;{\left(F^{\left(p\right)}u\right)}^{+};\;p=1,...,P \end{array}$$

where {·}_diag_ defines the vector composed of the principal diagonal of the embraced matrix, ⊙ defines the Shur-Hadamar (element by element) product and **F**^(^*^p^*^)^ represents the reconstructive/enhancement regularization technique, respectively.

To optimize the search of the SFO **F**, the following *DEDR* strategy [12] is formulated:

(2) $$F\to \underset{F}{\text{min}}\;\{ℜ(F)\}$$

where:

(3) $$ℜ(F)=\text{trace}\left\{(\mathrm{FS}-I)A{\left(\mathrm{FS}-I\right)}^{+}\right\}+\alpha \;\text{trace}\left\{{\mathrm{FR}}_{n}F^{+}\right\}$$

implies the minimization of the weighted sum of the systematic and fluctuation errors in the desired estimate **b̂**, where the selection (adjustment) of the regularization parameter *α* and the weight matrix **A** provide the additional experiment design degrees of freedom incorporating any descriptive properties of a solution if those are known a priori [12,20]. For more detailed information related in how to optimize the SFO **F**, see references [12,13].

Having established the optimal reconstructive RS estimators, let us now consider the way in which the processing of the data vector **u**, which results in the optimum estimate **b̂**, can be performed computationally. For this purpose, we refer to the estimator (1) as a multi-stage computational procedure. Also, from the algorithmic analysis, we outline the following important remarks regarding a possible hardware level architecture accelerator for complex reconstructive computational tasks required for implementing different RS imaging methods.

- First, the point spread matrix (PSMs) [12] operations of the SFO can be calculated in parallel over the azimuth and range axes can be calculated concurrently.
- Second, the Shur-Hadamar operation and the parallel reconstructive/enhancement operations of **F**^(*p*)^ **u** are able to be designed in a dual-core architecture. Notice that in Equation (1), the complex signal processing operations were algorithmically adapted for their efficient implementation.

#### 2.1.2. Computational Partitioning Process

In this subsection, it is presented how to perform an efficient HW/SW partitioning of the computational tasks. The aim of the partitioning problem is to find which computational tasks can be implemented in an efficient hardware architecture looking for the best trade-offs among the different solutions [23,24]. The solution of the problem requires, first, the definition of a partitioning model that meets all the specification requirements (*i.e*., functionality, goals and constraints).

The proposed partitioning stage is clearly influenced by the target architecture onto which the HW and the SW will be mapped. We begin with the specifications of the system-level partitioning functions and detailing the selected design quality attributes for the HW/SW co-design aimed at the definition of the computational tasks that can be implemented in the dual super-systolic core form, namely: hardware area (*ha*), hardware execution time (*ht*), software execution time (*St*), and the selected system resolution (*n*); where *maxha*, *maxht* and *maxSt* represent the upper bounds of these constraints. In particular, for implementing the fixed-point RS estimator operations of Equation (1), the partitioning process must satisfy the following performance requirements [25].

- The system must always satisfy the constraints: 0 ≤ *ha < maxha*, 0 ≤ *ht < maxht*, for each *i*th hardware accelerator *Ac_i_, i* = 1,…,*l*; and 0 ≤ *St < maxSt*, for the DSP/embedded processor *E*. These parallel hardware accelerators {*Ac_i_*} and the DSP/embedded processor compose the target architecture *Target* = {*E*, *Ac_i_*, *n* }, for the pre-selected FPGA with the corresponding predetermined architecture constraints ***C***: {0 ≤ *ha < maxha*; 0 ≤ *ht < maxht*; 0 ≤ *St < maxSt*}.
- Each block implementation {*Bl* (*Ac_i_*), *Bl* (*E*)} must satisfy the predefined execution time performance requirements: *τ*{*Bl*(*Ac_i_*|*C_i_*); *i* = 1,…,*l*} and *τ*{*Bl*(*E*|**C***_E_*)} conditioned by the specified above architecture constraints {**C***_i_*: {0 ≤ *hti < maxhti*; 0 ≤ *hai < maxhai*}; *∀ i* = 1,…, *l*}, and **C***_E_*: 0 ≤ *St < maxSt*, correspondingly.

Now, the HW/SW co-design system architecture is to be optimized via bounding the total expected system processing time *τ* = *τ*{*Bl*(*Ac_i_*|**C***_i_*)}evaluated via:

(4) $$\tau \{\mathrm{Bl}(\mathrm{Ac}|C_{i})\}=(\underset{i}{\text{max}}\{\tau \{\mathrm{Bl}({\mathrm{Ac}}_{i}|C_{i})\}\}+\tau \{\mathrm{Bl}(E|C_{E})\})<T_{C++}$$

where T_C++_, represents the execution time required for implementing the corresponding RS-related regularization algorithms in the standard C++ computational environment.

Note that from the formal SW-level co-design point of view, such RS-regularized techniques, Equations (1–3) can be considered as a properly ordered sequence of the reconstructive signal processing operations that one next can perform in an efficient computational fashion using the proposed above HW/SW co-design paradigm.

#### 2.1.3. Architecture Design Procedure Using HPEC Techniques

Following the presented above partitioning paradigm, one can now decompose the fixed-point RS-regularized algorithms developed at the SW-design into the DSP/embedded processor and the specialized high-speed hardware accelerators *Ac_i_, i* = 1, …, *l*. In this study, the proposed bit-level dual super-systolic core is aggregated with a DSP/embedded processor via the proposed above HW/SW co-design, as illustrated in Figure 1.

In the design, the SSAs require high data bandwidth of data exchange with the DSP/embedded processor. Another challenging task of the co-design is how to manage the large block of data avoiding unnecessary data transfers from/to the embedded processor to/from the proposed bit-level HW accelerator.

The main parameters to consider in the partitioning stage are the task execution speed and the area required by its HW-level implementation. Based on those parameter considerations, the HW/SW co-design is carried out, which consists in deciding which tasks should be executed in SW and which one should be implemented by HW. Additionally, a number of different loop optimization techniques (*i.e*., loop optimization, loop unrolling, tiling, loop interchange, *etc*.) used in HPEC are implemented in order to exploit the maximum possible parallelism in the design (see [2], for more details). Also, the fixed-point software analysis stage (*i.e*., for this study is employed the selection of 9 bits integer and 23 fractional bits with rounding to nearest format for all the fixed-point operations) and the C/C++ reference implementation is realized. Such precision guarantees numerical computational errors less than 10^−5^ referring to the MATLAB Fixed Point Toolbox [22]. Remark that the RS acquired images are stored and loaded from a compact flash device, and the resulting enhanced images are also stored to the same memory device. Finally, the architecture in form of a dual SSA core may be implemented on Field Programmable Gate Arrays (FPGAs) or coarse-grained [26] programmable array architectures.

## 3. Dual Super-Systolic Array Core

**Figure 2.**
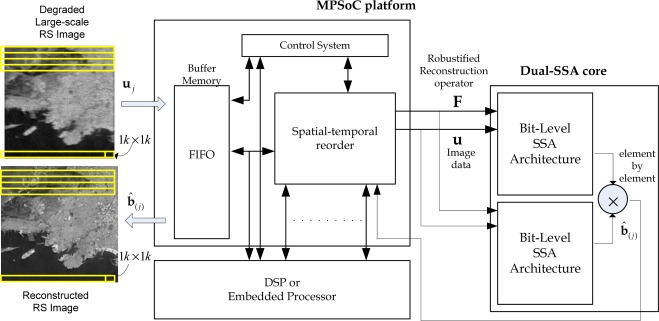
MPSoC platform of RS algorithms via the HW/SW co-design paradigm.

The super-systolic array (SSA) is a generalization of the systolic array (SA). It is a specialized form of an architecture, where the cells (*i.e*., processors), compute the data and store it independently of each other. SSAs consist of a network of cells (*i.e*., processing elements (PE)) in which each cell is conceptualized as another SA in a bit-level fashion. The SA architectures provide an optimal platform for the efficient HW-level implementation of an amount of reconstructive signal processing (SP) algorithms as coprocessor accelerators [27,28]. In this study, the implementation of a custom high-speed architecture, *i.e*., the dual SSA core, represents a new paradigm in the design of HPECs architectures which drastically reduce the processing time of the addressed reconstructive SP technique. Figure 2 presents a multiprocessor system on chip (MPSoC) platform for the enhancement/reconstruction of RS algorithms via the HW/SW co-design paradigm.

The first stage of the SSA-based design flow of Figure 2 consists in transforming the nested loop algorithms of the selected RS-reconstructive operations, in a parallel algorithmic representation with local and regular dependencies [27,28]. Next, with the tiling technique, the large-scale index space is divided into regular tiles (or blocks) of a real-size RS scene frame, and then traversing the tiles to cover the whole index space [27–29]. Finally, the dual SSA core is developed as a co-processor structure. The main challenge of this study is to present a methodology for the development of such dual SSAs core from the addressed reconstructive signal processing operations and also for the generation of the efficient control system. This is one of the major contributions of this paper due the lack of HPEC tools and also due the lack of control system methodologies.

**Figure 3.**
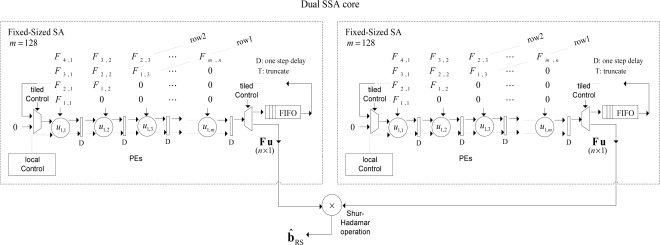
Dual SSA core of the RS-related estimator.

In Figure 3, the conceptualization of the fixed-point dual SSA core is depicted. From the analysis of Figure 3, one can deduce the dual SSA machine running in parallel, and then, the element by element Shur-Hadamar operation, for the implementation of the optimal reconstructive RS estimator of Equation (1). This SSA efficiently computes the complex SSP estimation of the RS algorithms. Notice that at this implementation stage, Figure 3 only describes the HW-level architecture at a coarse grain detail. Through this figure, one also can deduce how such complex matrix operators are working in order to perform optimal reconstructive RS estimator.

**Figure 4.**
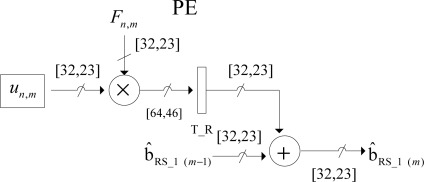
MAC operation of each PE.

Both SSA architectures perform the discrete-form representation of the desired spatial spectrum pattern (SSP) in a high-performance structure. The multiply-accumulate (MAC) operation implemented in each processing element (PE) is now depicted in Figure 4.

The internal structure of each PE presented in Figure 4 contains a multiplier and an adder. Each PE receives 32-bits operands and generates 64-bits product. Then, the product is truncated and then, rounded into 32-bits using a nearest rounding scheme with a fixed-point adopted representation of 9 integers and 24 decimals. The bit-level SSA representation of this MAC module will be presented further on in Section 3.4.

### 3.1. Parallel Algorithm Transformation

The algorithm of the selected RS-reconstructive operations, *i.e*., the **b̂** = (**FU**) ⊙ (**FU**)^+^ can be represented by nested loops or FOR-loops programs. First, let us define from the reconstructive RS estimator of Equation (1), the *n* × *m* matrix **F** and the vector **u** of dimension *m* as follows:

(5) y=Fu

where **y** is an *n-*dimensional (*n*-*D*) output vector. The *j-th* element of **y** is computed as:

(6) $$y_{j}=\sum_{i=1}^{n}F_{\mathrm{ji}}u_{i},\;\;\;\;j=1,...,m$$

where *F_ji_* represents the corresponding element of **F**.

Next, the localization method converts the algorithm into an algorithmic representation with local and regular dependencies [27,28]. The following algorithm achieves locality via affine scheduling transformations as presented below:

(7) $$\begin{array}{l} \mathrm{input operations}\\ \begin{array}{ll} F[i,\;j]\leftarrow F_{i,j-1} & \forall (i,\;j)\;\;|\;\;1\leq i\leq n;\;\;1\leq j\leq m.\\ u[0,\;j]\leftarrow u_{j} & \forall \;\;j\;\;|\;\;1\leq j\leq m.\\ y[i,0]\leftarrow 0 & \forall \;\;i\;\;|\;\;1\leq i\leq n. \end{array}\\ \;\mathrm{computations}\\ \;\mathrm{for}(i=0;i<n;i++)\{\\ \;\;y[i,0]=0;\\ \;\;\mathrm{for}(j=0;j<m;j++)\{\\ \;\;\;u[i,\;j]=u[i-1,\;j];\\ \;\;\;y[i,\;j]=y[i,\;j-1]+F[i,\;j]\cdot u[i,\;j];\;\;\;\}\;\}\\ \mathrm{output operations}\\ \begin{array}{ll} y[i]\leftarrow y[i,m] & \forall (i,\;j)\;\;|\;\;1\leq i\leq n;\;\;\;1\leq j\leq m \end{array}.\\ \mathrm{where the index space is}\\ I=\{{\left(i,\;j\right)}^{T}\in {\mathbb{Z}}^{2}|1\leq i\leq n;\;\;1\leq j\leq m\} \end{array}$$

Once the algorithm is transformed into their localized representation (*i.e*., locally recursive form), one is ready to proceed with the tiling procedure in order to achieve realistic large-scale RS structures with the fixed-sized SA architecture.

### 3.2. Tiling Transformation Technique

The tiling technique is a well known loop transformation used to automatically create sub-block algorithms [26,29,30]. The advantage of this method is that, while computing within a block, there is a high degree of data locality, allowing better performance. The *tiling* procedure consist of dividing the large-scale index space defined by the loop structures into regular tiles (or blocks) of some real-scale size and shape RS complex operations, and then traversing the tiles to cover the whole index space [30]. The conventional *tiling* procedure combine two well-known transformations: loop permutation and strip-mining. The loop permutation is used to establish the order in which the iterations inside the tiles are traversed and the strip-mining transformation is used to partition one dimension of the index space into strips. It also decomposes a single loop into two nested loops; the outer loop steps between strips of consecutive indexes, and the inner loop traverses the indexes within a strip. Both transformations can be obtained using the theory of unimodular transformations and, to compute the exact bounds, the Fourier-Motzkin elimination algorithm [30] is applied.

Now, considering the locally recursive representation presented in Equation (7), the strip-mining transformation is applied to the outermost loop in order to perform the one-dimensional partition of the *i*-index algorithm. The resulting index partition is represented as follows:

(8) $$\begin{array}{lll} \mathrm{for}(i=0;i<n;i++) & & \mathrm{for}(\mathrm{tile\_i}=0;\mathrm{tile\_i}<n;\mathrm{tile\_i}=\mathrm{tile\_i}+\mathrm{StSizei})\\ \mathrm{for}(j=0;j<m;j++) & \overset{\text{strip}-\text{mining}}{\to } & \mathrm{for}(i=\mathrm{tile\_i};i\leq \text{min}[\mathrm{tile\_i}+\mathrm{StSizei}-1,n-1\;];i++)\\ \mathrm{Loop body} & & \mathrm{for}(j=0;j<m;j++)\\ & & \mathrm{Loop body}\;\;[i,\;j] \end{array}$$

where *tile_i* represents the *i*-index-tile loop, *i* and *j* are the inner element’s loops and *StSize* is the strip size.

The second step of the tiling procedure consists in implement the loop permutation transformation based on the Polytope model [30]. For the loop permutation, the following unimodular transformation $T_{P}=\left[\begin{array}{cc} 0 & 1\\ 1 & 0 \end{array}\right]$ was applied in order to permute the index-space of the locally recursive algorithm **I** = [*i j*]^T^ into the required new index-space **I_P_** = [*i’ j’*]^T^=[*j i*]^T^. In this step, it is defined the Polytope model as a set of inequations such as **ΓI_P_** ≤ **H**, where **I_P_** = [*i’ j’*]^T^ represents the index-space after the *i*-strip-mining procedure, and the matrix **Γ** and vector **H** represents the boundaries of each FOR-loop of the algorithm presented above in Equation (8).

The source Polytope is described in a convex form by a set of half-spaces, where the intersection of all half-spaces corresponds to the Polytope and the target representation is presented as follows:

(9) $$\begin{array}{c} \Gamma \;I\leq H\\ T_{P}\;I=I_{P} \end{array}\}\;\;\Rightarrow \;\begin{array}{c} \Gamma \;T_{P}^{-1}\;I_{P}\leq H\\ \Gamma I_{P}\leq H \end{array}\;\Rightarrow \;\underset{\Gamma }{\underset{︸}{\left[\begin{array}{cc} -1 & 0\\ 1 & 0\\ 1 & 0\\ 0 & -1\\ 0 & 1 \end{array}\right]}}\;\underset{T_{P}^{-1}}{\underset{︸}{\left[\begin{array}{cc} 0 & 1\\ 1 & 0 \end{array}\right]}}\;\underset{I_{P}}{\underset{︸}{\left[\begin{array}{c} i\prime \\ j\prime \end{array}\right]}}\;\leq \;\underset{H}{\underset{︸}{\left[\begin{array}{c} -\mathrm{tile\_i}\\ \mathrm{tile\_i}+\mathrm{StSize}-1\\ n-1\\ 0\\ m-1 \end{array}\right]}}.$$

The new loop bounds are derived with the Fourier-Motzkin elimination algorithm. The resulting reconstructive RS algorithm after the loop permutation transformation is now presented, in which the proper substitutions are integrated:

(10) $$\begin{array}{l} \mathrm{for}(\mathrm{tile\_i}=0;\mathrm{tile\_i}<n;\mathrm{tile\_i}=\mathrm{tile\_i}+\mathrm{StSizei})\{\\ \mathrm{for}(j=0;j<m;j++)\{\\ \mathrm{for}(i=\mathrm{tile\_i};i\leq \text{min}[\mathrm{tile\_i}+\mathrm{StSizei}-1,n-1\;];i++)\{\\ \mathrm{Loop body}\;\;[j,\;i\;]\\ \}\} \end{array}$$

The third step of the tiling procedure corresponds again to the strip-mining transformation procedure but in this case, the procedure is applied over the *j*-index. Furthermore, the resulting tiled algorithm after this strip-mining transformation is next represented. The final step consists in to employ again the loop permutation. This final transformation is required to order the inner loop for the final tiled algorithm represented as follows:

(11) $$\begin{array}{l} \mathrm{for}(\mathrm{tile\_i}=0;\mathrm{tile\_i}<n;\mathrm{tile\_i}=\mathrm{tile\_i}+\mathrm{StSizei})\{\\ \mathrm{for}(\mathrm{tile\_j}=0;\mathrm{tile\_j}<m;\mathrm{tile\_j}=\mathrm{tile\_j}+\mathrm{StSizej})\{\\ \mathrm{for}(i=\mathrm{tile\_j};i\leq \text{min}[\mathrm{tile\_j}+\mathrm{StSizej}-1,m-1];i++)\{\\ \mathrm{for}(j=\mathrm{tile\_i};j\leq \text{min}[\mathrm{tile\_i}+\mathrm{StSizei}-1,n-1];j++)\{\\ \;\;\mathrm{Loop body}\;\;[i,\;j]\\ \;\;\}\} \end{array}$$

From the analysis of the tiled parallel algorithm of Equation (11), one is ready to deduce the dual SSA core and the local control system architecture presented in Figure 2.

### 3.3. Space-Time Mapping Onto Fixed-Sized SAs

The space-time mapping procedure onto SAs is a technique that transforms an index-space representation into a space-time representation where each node of their iteration node is mapped to a certain PE and it is scheduled to a certain instance of time [27,28]. Recall that the SA is a space-time representation of the computational operations, in which the function description defines the behavior within a node, whereas the structural description specifies the interconnections (edges and delays) between the corresponding graph nodes [27]. In order to derive a SA architecture with a minimum possible number of nodes, we address a linear projection approach for processor assignment, *i.e*., the nodes of the structure array in a certain straight line are to be properly projected onto the corresponding PEs of the SA represented by the corresponding assignment projection vector **d**. Thus, we seek for a linear order reduction, in which the transformation **T_m_** : **G***^N^* → **Ĝ**^*N*−1^ maps the *N*-dimensional dependence graph (**G***^N^*) onto the (*N*−1)-dimensional SA (**Ĝ**^*N*−1^), where *N* represents the dimension of their dependence graph (see proofs in [19,27] and details in [30]). Moldovan in [31], proved the mapping theory, as follows:

(12) $$T_{m}=\left[\begin{array}{c} \Pi \\ \Sigma \end{array}\right],$$

where **Π** is a (1 × *N*) − *D* vector (composed of the first row of **T_m_**) which (in the partitioning terms [19,29]) determines the time scheduling, and the (*N* − 1) × *N* sub-matrix **Σ** in Equation (29) is composed of the rest rows of **T_m_** that determine the space processor specified by the so-called projection vector **d** [19,31]. Next, such partitioning (12) yields the regular SA of (*N* – 1) – *D* specified by the mapping:

(13) $$T_{m}\;\Phi =K,$$

where **K** is composed of the new revised vector schedule (represented by the first row of the SA) and the inter-processor communications (represented by the rest rows of the SA), and the matrix **Φ** specifies the data dependencies of the parallel representation algorithm. For a more detailed explanation of the mapping theory, see [19,27,28]. Next, we define the following specifications for performing the mapping of the fixed-sized reconstructive RS operation, *i.e*., the **y** = **Fu** algorithm of Equation (1), onto each parallel SA core: **Π** = [1 1] specifies the vector schedule, **d** = [1 0] specifies the projection vector, and **Σ** = [0 1] specifies the corresponding space processor.

With these specifications, the transformation matrix becomes $T=\left[\begin{array}{c} \Pi \\ \Sigma \end{array}\right]=\left[\begin{array}{cc} 1 & 1\\ 0 & 1 \end{array}\right]$. Next, we specify the dependence vectors of the locally recursive algorithm: $\Phi =[\begin{array}{lll} \Phi_{F} & \Phi_{u} & \Phi_{y} \end{array}]$, where $\Phi_{F}=\left[\begin{array}{c} 0\\ 1 \end{array}\right]$, $\Phi_{u}=\left[\begin{array}{c} 1\\ 0 \end{array}\right]$ and $\Phi_{y}=\left[\begin{array}{c} 0\\ 1 \end{array}\right]$ represent the dependencies of the corresponding variables in the algorithm. These specifications result in the following SA dependencies:

(14) $$T\Phi =K\;\to \;\;\underset{T}{\underset{︸}{\left[\begin{array}{cc} 1 & 1\\ 0 & 1 \end{array}\right]}}\;\underset{\Phi }{\underset{︸}{\left[\begin{array}{ccc} 0 & 1 & 0\\ 1 & 0 & 1 \end{array}\right]}}=\underset{K}{\underset{︸}{\left[\begin{array}{ccc} 1 & 1 & 1\\ 1 & 0 & 1 \end{array}\right]}}.$$

The number of PEs required by each coarse grain SA of the dual SSA core architecture is *n*, and the required computational time is 2*n* – 1 clock periods. In Figures 3 and 4, how the SA architecture is implemented at a coarse grain detail for the reconstructive processing of realistic large-scale RS scenes (e.g., 1*K* × 1*K* pixel size) by reusing the same fixed-sized SA architecture for each partitioned scene frame is conceptualized. At this stage, the scalability in terms of HW resources can be analyzed varying the number of PEs of the fixed-sized SA architecture.

### 3.4. Bit-Level Fixed-Sized Dual SSAs Core

**Figure 5.**
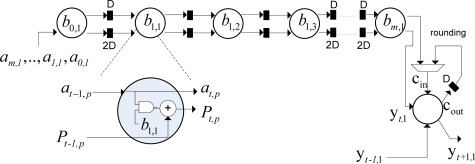
Bit-level SSA of the MAC structure.

Once the coarse grain SAs of the selected RS reconstructive algorithm have been defined, we are ready to conceptualize and implement the bit-level fixed-sized dual SSA core. The internal structure of each fixed-sized SA contains identical PEs linearly-connected also in a systolic fashion. The same SA-based design flow, implemented in the previous sub-sections (*i.e*., algorithmic implementation, tiling and mapping techniques), is again employed for the bit-level dual SSA core as an accelerator structure. Figure 5 illustrates the fixed-sized bit-level SSA architecture (*i.e*., at a fine grain detail) of each PE of the previously conceptualized RS reconstructive operation.

From the analysis of Figure 5, one can note the improvement achieved with this highly-pipelined architecture in terms of its hardware performance. The bit-level multiply accumulate (MAC) structure of each PE is described as follows: the architecture receives 32-bits operands and generates 64-bits product. The multiplexor in the figure performs the truncate function of the bit-level MAC operation implemented by the array of logic cells. Finally, the logic full-adder implements the rounded function for a better performance.

The SA for performing the bit-level MAC operation of each PE of the dual SSA core employs the following specifications in the transformation defined by Equation (12): **Π** = [1 2] specifies the vector schedule, **d** = [1 0] specifies the projection vector and **Σ** = [0 1] specifies the corresponding space processor. The dependence matrix of the MAC algorithm is specified by $\Phi =\left[\begin{array}{ccc} 1 & 0 & -1\\ 0 & 1 & 1 \end{array}\right]$. For the mapping-optimized projection vector **d** = [1 0], these specifications yield the following SA structure:

(15) $$T\Phi =K\;\to \;\left[\begin{array}{cc} 1 & 2\\ 0 & 1 \end{array}\right]\;\left[\begin{array}{ccc} 1 & 0 & -1\\ 0 & 1 & 1 \end{array}\right]=\left[\begin{array}{ccc} 1 & 2 & 1\\ 0 & 1 & 1 \end{array}\right].$$

This bit-level SA-based MAC architecture requires an array of *ρ* bit-level multiply-accumulate operations with 3*ρ* − 2 clock periods. In this study, we consider 32 bits operands (*i.e*., *ρ* = 32). The high-performance analysis achieved with this dual SSA core architecture will be presented further on in Section 4.

## 4. Implementation Results

In this section, the results of the hardware-level implementation of the reconstructive complex RS functions with the employment of a high-speed fixed-sized dual SSA core accelerator are reported. The addressed architecture drastically reduce the computationally load of the enhancement/reconstruction real-world Geospatial images acquired with different fractional multisensory SAR systems. In order to demonstrate the best area-time trade-off of the digital implementation and the high accuracy of the proposed RS hardware accelerator, the authors have conducted some test-case scenarios of the real-world RS images characterized by the point spread function (PSF) of a Gaussian “bell” shape in both directions of the 2-D scene (in particular, of 16 pixel width at 0.5 from its maximum for the 1*k*-by-1*k* BMP pixel-formatted scene) with the selected FPGA target platform.

### 4.1. Architecture Performance Analysis

The comparative performance analysis of the HW-level implementation of the dual SSA core architecture is presented in this sub-section. Such HW-level performance analysis is focused on define which is the best area-time tradeoff. The Xilinx XST tool of the Integrated Software Environment (ISE™) WebPACK™ was used for the synthesis of the proposed architecture. The clock frequency of 100 MHz is the selected timing constrain considered for the synthesis procedure. The following parameters were considered in the synthesis: the order of each fixed-sized SSA is *m* = 64 and the data-output sample word length of 32 bits. In Table 1, it is reported the synthesis results evaluated by different metrics that are indicative of the efficiency of the proposed reconfigurable architecture with respect to the selected FPGA-targets Xilinx Virtex-5 XC5VFX130T and Virtex-4 XC4VSX35.

From the analysis of Table 1, one can conclude that one of the relevant implementation results is related to the high-speed and high-throughput performance, in which the proposed architecture is able to run up to 920.93 MHz.

**Figure 6.**
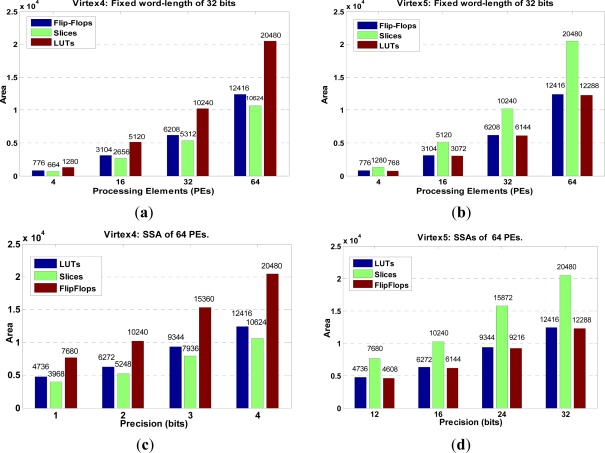
HW-resource scalability analysis: (**a**) varying the PEs for Virtex-4, (**b**) varying the PEs for Virtex-5, (**c**) varying the bits precision for Virtex-4 and (**d**) varying the bits precision for Virtex-5.

Next, the scalability analysis in terms of HW resources is presented for the relevant dual SSA core architecture in Figure 6.

In such analysis, the number of precision bits and the number of processing elements (PEs) are modified in order to estimate the HW resources. The results reveal the area resource utilization of the dual SSA-based architecture in the FPGA-target platform.

Other alternative implementations for RS applications that implement specialized HW architectures (*i.e*., SA-based) are presented in [16,17,32,33]. For example, in [32], a digital custom space-based FPGA co-processor for high-performance space computing is presented. However, in the design of such coprocessors do not consider an MPSoC scheme for a parallel real-time application. Another approach for high-speed computational implementation of reconstructive RS image processing based on the use of clusters of PCs was presented by Yang *et al*. in [33], in which the cluster NSPO Parallel TestBed for performing parallel radiometric and geometrical corrections of the large-scale 3,600 × 2,944-pixel RS images was implemented. The reconstructive image processing was conducted using a PC-Cluster composed by three PCs each one with a Pentium-III 550 MHz with 128 MB of RAM connected with 100 Mbps Fast-Ethernet LAN. The processing time achieved with such three-PC cluster was only 33.3 s (near-real time for conventional RS users), while the corresponding processing performed with one single processor required 84.65 s. Once more, the authors believe that this dual SSA core is unique and completely differs from other specialized HW architectures in recent RS applications.

In the next section, a concrete real-world Geospatial test application from multi-sensor array SAR systems scenario is presented. This test will evaluate the accuracy of the proposed dual SSA core that it is integrated in a MPSoC design via the HW/SW co-design. The reported results of such enhancement/reconstructive model will be also discussed further on in the next sub-section.

### 4.2. High-Resolution Enhancement/Reconstruction of RADAR/SAR Imagery: A Test Case Study

In this sub-section, we present an illustrative test case study related to the HW-implementation of the Weighted Constrain Least Square (WCLS) regularization technique for the enhancement/reconstruction of RS applications. This HW-implementation is based on the proposed here, dual SSA core in aggregation with a Microblaze embedded processor and the On Chip Peripheral Bus (OPB) for transferring the data to/from the embedded processor. In the HW design, we consider to use the precision of 32 bits fixed-point, 9-bit integer and 23-bits decimal for the implementation of all fixed-point operations in each SSA core. Once the HW/SW co-design methodology has been employed, we are ready to establish the verification statements to evaluate the accuracy of the MPSoC system.

In the HW implementation, a large scale (1*K*-by-1*K*) pixel format RS image borrowed from the real-world high-resolution terrain SAR was employed. The quantitative evaluation of the RS reconstruction performances was employed using the following quality metric defined by the improvement in the output signal-to-noise ratio (IOSNR) [34]. In this evaluation, the signal formation operator of all RS images is factorized along two axes in the image plane: the azimuth (horizontal axis, *x*) and the range (vertical axis, *y*). Following the common practically motivated technical considerations [3,4,15–20], we modeled the Gaussian shape in the SAR range PSF *Ψ_r_*(*y*) in the range direction *y*, and the side-looking SAR azimuth PSF *Ψ_a_*(*x*) in the cross-range direction *x* at the zero crossing level for the simulated SAR system with fractionally synthesized aperture.

The quantitative measures of the image enhancement/reconstruction performance gains achieved with the particular employed WCLS technique, evaluated via the IOSNR metric, are reported in Table 2 with two different real-world high-resolution scene images.

**Figure 7.**
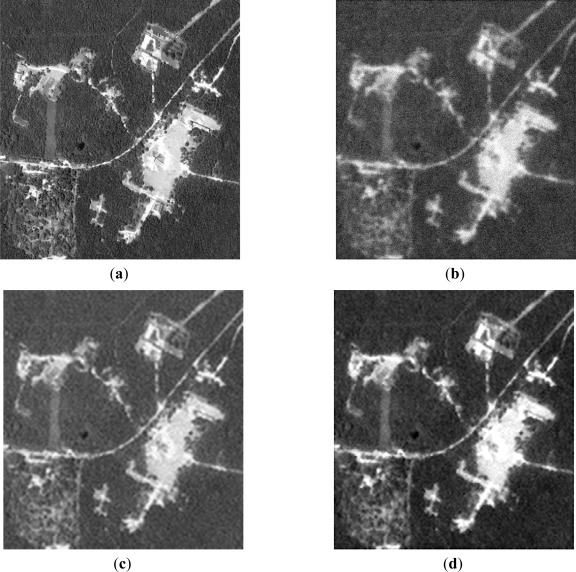
Implementation results for the first observation scenario: (SNR μ = 10 dB): (**a**) Original tested scene; (**b**) degraded scene image formed applying the MSF method; (**c**) image reconstructed applying the CLS algorithm; (**d**) image reconstructed applying the WCLS algorithm.

**Figure 8.**
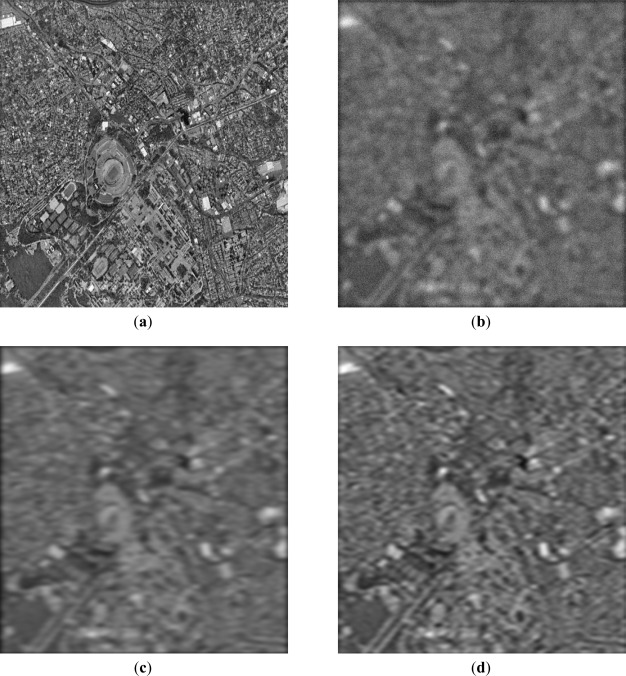
Implementation results for the second observation scenario: (SNR μ = 10 dB): (**a**) Original tested scene; (**b**) degraded scene image formed applying the MSF method; (**c**) image reconstructed applying the CLS algorithm; (**d**) image reconstructed applying the WCLS algorithm.

Next, the qualitative results are presented in Figures 7 and 8, with two different real-world high-resolution scenes. Figure 7(a,b) show the original test scene images. Figures 7(b) and 8(b) present the noised low-resolution (degraded) scene images formed with the conventional MSF algorithm. Figures 7(c) and 8(c) present the scene images reconstructed with the CLS-regularized algorithm. Figures 7(d) and 8(d) present the scene images reconstructed employing the WCLS-regularized algorithm.

From the analysis of the qualitative and quantitative implementation results reported in Figures 7 and 8, and Table 2, one may deduce that the dual SSA core was efficiently integrated in MPSoC embedded system via the HW/SW co-design method. Additionally, such WCLS implementation results over-perform the robust non-adaptive CLS in all simulated scenarios.

Finally, in Table 3, we report the processing times required for implementing the WCLS image reconstruction algorithms using the developed dual SSA core in a MPSoC embedded system.

In the first case in Table 3, the WCLS algorithm was implemented in C++ software in a personal computer (PC) running at 3 GHz with a AMD Athlon (tm) 64 dual-core processor and 2 GB of RAM memory. In the second case, the same WCLS-related algorithm was implemented using the proposed here efficient architecture approach with the specialized dual SSA core employed in the Xilinx FPGA Virtex-5 XC5VFX130T.

The implementation of this high-speed architecture helps to drastically reduce the overall processing time. Particularly, the proposed implementation of the WCLS algorithm with the proposed HW-specialized architecture takes only 0.81 s for the large-scale RS image reconstruction in contrast to 12.6 s required with the C++ reference implementation. Thus, the processing time of the proposed dual SSA core-oriented architecture is approximately 16 times less than the corresponding processing time achievable with the conventional C++ PC-based implementation.

In this regard, the emergence of specialized hardware devices such as the dual SSA core in FPGAs represents a new paradigm to develop real-time systems for remote sensing data processing. The increasing computational demands of remote sensing applications can now be benefit from these compact hardware components, taking advantage of the small size and relatively low cost of these units as compared to clusters or networks of computers. These aspects are of great importance in other areas like hyperspectral imaging for Earth observation and remote sensing missions that require an extremely large number of spectral bands and high spatial resolution.

## 5. Conclusions

In this study, a high-speed dual SSAs core which accelerates complex regularization operations for the real-time enhancement/reconstruction of large-scale RS imaging of radar/SAR sensor systems is presented. Also, the design methodology for real time implementation of such specialized arrays of processors using high performance embedded computing (HPEC) architecture was developed.

The dual SSA core was evaluated as follows: the architecture was aggregated with a Microblaze embedded processor in MPSoC structure via the HW/SW co-design paradigm. The WCLS regularization method was algorithmically adapted (using parallel computing techniques) and implemented in a real time computational mode (the ‘real-time’ being understood in a context of conventional RS users). The performance analysis and the qualitative and quantitative results reveal that the dual SSA core as accelerator units drastically increase the throughput and the processing time of large-scale real-time image processing requirements while performing the reconstruction of real-world hyperspectral RS imagery. In addition, the authors believe that the FPGA/DSP-based systems in aggregation with novel bit-level super-systolic architectures offer enormous computation potential in RS systems for newer Geospatial applications.

## Figures and Tables

**Table 1.** Synthesis results of the proposed dual SSA core. SSA order: *m* = 64.

***Device*→**	***Virtex-4 XC4VSX35***	***Virtex-5 XC5VFX130T***

LUTs	12,416	12,416
Slices	10,624	20,480
Flip-Flops	20,480	12,288
Output bit-width	32	32
Max. Clock freq. (MHz)	910.47	920.93

**Table 2.** IOSNR of the WCLS algorithm evaluated for different SNRs.

**SNR [dB]**	**FIRST SCENARIO *Ψ_a_*(*x*) = 13**		**SECOND SCENARIO *Ψ_a_*(*x*) = 25**	

	**IOSNR^(CLS)^ [dB]**	**IOSNR^(WCLS)^ [dB]**	**IOSNR^(CLS)^ [dB]**	**IOSNR^(WCLS)^ [dB]**

5	2.12	3.26	2.67	3.92
10	3.43	4.45	4.59	5.83
15	4.17	5.23	5.51	7.64
20	5.36	6.82	6.47	9.87
25	6.94	8.27	8.32	11.16

**Table 3.** Processing times required for implementing the WCLS algorithm.

**Implementation →**	**Processing time (s)**
	**WCLS**
PC-Oriented Implementation of the WCLS	12.6
Implemented with the proposed dual SSA core architecture	0.81
